# Relaxation in action: enhanced vagal modulation during *Virabhadrasana* (a standing yoga-asana) despite orthostatic demand suggests efficient regulation of effort

**DOI:** 10.3389/fnhum.2026.1887856

**Published:** 2026-09-15

**Authors:** Leena Phadke, Reinhard Boegle, S. N. Bhavsar, Sanjay Phadke

**Affiliations:** 1 SKN Medical College, Pune, India; 2 Yoga Forum, Munich, Germany; 3 Centre for Behavioral Medicine, Pune, India; 4 Deenanath Mangeshkar Hospital and Research Centre, Pune, India; 5 Jehangir Hospital, Pune, India

**Keywords:** adaptive flexibility, central autonomic network, effort regulation, embodied regulation, heart rate variability, motor control, systems neuroscience, *Yoga-Āsana*

## Abstract

**Introduction:**

Classical *Yoga-Āsana* emphasizes not merely adoption of posture, but progressive reduction of unnecessary effort while maintaining stable engagement. However, the neurophysiological implications of effort modulation during yoga practice remain insufficiently understood. This study examined whether externally similar *Yoga-Āsana* performed with different effort organization may engage distinct autonomic regulatory organizations.

**Methods:**

Eight experienced female yoga practitioner-teachers (median age: 58 years; median yoga experience: 19 years) participated in a within-subject exploratory study. Heart rate variability (HRV) was assessed during three 5-min conditions: free-standing, effort-reduced “yogic” *Virabhadrasana* (Y), and *Virabhadrasana* (E) performed without deliberate effort reduction. HRV indices were analysed using Friedman and Wilcoxon signed-rank tests with Holm–Bonferroni correction.

**Results:**

Compared with *Virabhadrasana* (E), yogic *Virabhadrasana* (Y) demonstrated significantly increased RR interval, HF power and HF (nu), and significantly reduced LF (nu) and LF/HF ratio indicating preserved vagally mediated autonomic flexibility despite similar orthostatic demand and active postural engagement. DFAα1 values were significantly lower in the yogic condition.

**Discussion:**

This study provides preliminary proof-of-concept that externally similar but effortfully different postural behaviors (*Virbhadrasana* Y and E) were associated with different HRV patterns in a small group of experienced practitioners. Skilled yogic performance appears to involve efficient embodied regulation characterized by reduced regulatory burden, preserved adaptive flexibility, and relaxation within sustained action despite continued postural demand. These findings support the broader hypothesis that physiological cost depends not merely on task demands, but on the organization of regulation through which action is achieved.

## 1 Introduction

Yoga has gained worldwide popularity due to its broad range of reported health benefits, stimulating intensive neuroscience research into its underlying mechanisms. However, despite decades of investigation, major reviews repeatedly note substantial conceptual and methodological limitations in yoga research. A recent 2026 review concluded that evidence regarding the impact of yoga on brain structure and function remains inconsistent due to widespread methodological issues. That review explicitly recommended the necessity to “clarify the definition of yoga and to differentiate between the different components of yoga for the benefit of future studies” (Stoelers et al., 2026). Earlier major reports similarly emphasized the absence of a common theoretical framework (Ospina et al., 2007). Together, these observations point to a critical need to revisit the foundational operational principles of yoga practice itself.

The *Yoga Sutra of Patañjali*, widely regarded as the foundational text of classical yoga philosophy, provides precise operational descriptions of *Aṣṭāṅga Yoga* (Larson, 2018). This includes *Yoga-Āsana* (yogic postures), which remains the most widely practiced and scientifically studied component of yoga. The *Yoga Sutra* strictly defines the quality and internal organization of *Āsana* practice. *Yogasutra 2.46* describes *āsana* as characterized simultaneously by stability (*Sthira*) and ease (*Sukham*). *Yogasutra 2.47* identifies progress from effortfulness to effortlessness (*prayatna-śaithilya*) as the very crux of sustaining a posture, which subsequently drives the development of adaptability to the opposites (*dvandva*; *Yogasutra 2.48*). In its classical form, *Yoga-Āsana* involves intentional, sustained postures requiring continuous multimodal proprioceptive awareness, interoceptive monitoring, and controlled neuromuscular adjustment (Schmalzl et al., 2015). Crucially, it demands the progressive reduction of unnecessary cognitive and muscular effort. Postural stability is achieved not through rigid, forceful contraction, but through precise alignment and dynamic regulation that preserves natural breathing. Such skill typically requires prolonged training and refinement.

Although classical yoga emphasizes that action depends less on *what* is performed and more on *how* it is regulated, contemporary neurophysiological research has paid little attention to the internal organization of effort during practice. In motor behavior and cognitive neuroscience, effort is traditionally conceptualized as the allocation of physiological and cognitive resources required to meet task demands (Shenhav et al., 2017). In this framework, increasing task difficulty is linearly associated with greater recruitment of control processes and metabolic activation. Such formulations, however, emphasize the total *magnitude* of recruitment rather than the *efficiency* of regulation. Conversely, growing evidence from motor control and systems neuroscience suggests that skilled performance involves the optimization, rather than the maximization, of control (Harris and Wolpert, 1998; Todorov and Jordan, 2002; Latash et al., 2002). Expert action across domains is frequently characterized by economical movement organization, reduction of unnecessary muscular co-contraction, lower metabolic cost, and the preservation of adaptive physiological flexibility despite maintained task performance (André et al., 2019).

This distinction is highly relevant to yoga practice, where the exact same external posture can be achieved through markedly different internal control strategies. One mode may involve forceful muscular contraction without deliberate effort modulation, resulting in rigid stabilization and heightened corrective control. Another mode may deliberately minimize unnecessary effort through refined postural organization. Although these two modes appear externally identical biomechanically, they may reflect fundamentally different physiological states and central regulatory architectures. Importantly, this efficiency does not represent passive relaxation or fully automatic subcortical habit. Instead, it reflects a refined regulatory organization involving economical, real-time cortical modulation capable of minimizing control burden while preserving active task engagement. A systematic review which compared different styles of yoga (ashtanga, Bikram, gentle, hatha, Iyengar, power and vinyasa) indicates that metabolic and heart rate responses vary across yoga styles and *asana* (Forseth and Hunter, 2020).

Heart rate variability (HRV), a quantitative measure of cardiac autonomic modulation, provides an ideal non-invasive window into these subtle differences in central regulatory organization during ambulatory motor activity. Initially applied in cardiology research for prognostication (Kleiger et al., 1987), the value of HRV is now well established as a marker of health and allostatic load in prediction of morbidity and mortality (Jarczok et al., 2022; Phadke et al., 2020; Wadhokar et al., 2024). HRV reflects dynamic interactions among autonomic, respiratory, cardiovascular, and central regulatory systems. Contemporary neurovisceral integration models recognize that HRV indices reflect broader properties of systemic physiological organization rather than isolated autonomic “tone” (Shaffer and Ginsberg, 2017). Higher vagally mediated variability and preserved physiological complexity are generally associated with greater adaptability, resilience, and efficient self-regulation. Conversely, reduced variability and rigidified, over-controlled physiological dynamics are hallmark signatures of stress, chronic strain, and impaired regulatory capacity (Thayer et al., 2009).

The present study was motivated by two complementary objectives. The primary objective was to empirically examine, via linear and non-linear autonomic dynamics, the foundational operational principle that skilled yogic performance involves the progressive reduction of unnecessary effort (*prayatna-śaithilya*) during sustained task engagement. A secondary, broader objective was to explore the implications of these distinctions for the neuroscience of effort and adaptive regulation—specifically, whether efficient task performance can involve a reduction in central regulatory cost and the preservation of physiological flexibility rather than increased sympathetic activation alone.

To achieve this, autonomic dynamics were compared during three modes of standing performance: free-standing baseline, deliberately effort-reduced “yogic” performance of *Virabhadrasana* (Y), and *Virabhadrasana* (E) performed without deliberate effort reduction. Utilizing time-domain, frequency-domain, and non-linear fractal HRV measures, we examined whether externally similar standing postural tasks are associated with distinct physiological control strategies reflecting differential regulatory economy and adaptive flexibility.

## 2 Methods

### 2.1 Participant characteristics

Eight highly experienced, healthy female yoga practitioner-teachers were recruited for this within-subject, exploratory study (Median Age = 58 years; Median Yoga Experience = 19 years). All participants were extensively trained in classical *Yoga-Āsana* practice, practiced regularly, and were actively engaged in yoga instruction. All participants were accustomed to sustaining *Yoga-Āsana* postures for a minimum of 5 minutes, satisfying methodological requirements for frequency-domain HRV analysis using fast Fourier transform (FFT)-based spectral estimation. Skilled subjects were deliberately enrolled in this exploratory study because of their ability to modulate the effort at will to test the two conditions involving different effort. Exclusion criteria included any self-reported history of cardiovascular disease, autonomic neuropathy, psychiatric disorders, or the use of medications known to alter cardiovascular autonomic function (e.g., beta-blockers). Participants were instructed to abstain from caffeine, alcohol, and strenuous physical exercise for at least 24 h prior to testing. Baseline ECG recordings were obtained to exclude cardiac conduction abnormalities. All participants were normotensive, and within normal BMI range.

The study protocol was approved by the Institutional Ethics Committee of SKNMC and GH, and all participants provided written informed consent before commencing the experimental protocol.

### 2.2 Experimental design and protocol

A within-subject, repeated-measures design was employed to compare cardiac autonomic profiles across three distinct, consecutive 5-min conditions following initial period of supine rest. As the ANS response is affected by many stimuli, including physical activity prior to the experiment, a resting supine period was included to minimize any spillover effect and ensure uniformity at the start of the study. The conditions were sequenced as follows to preserve experimental safety and steady-state:

1 *Free-standing baseline*: Participants stood quietly in a relaxed, anatomical posture. This condition served as a standard orthostatic challenge and an active-standing reference baseline.

Performance of “Yogic” *Virabhadrasana* (Y) and *Virabhadrasana* (E) followed.

2 *Yogic Virabhadrasana (Y)*: Performance of an *āsana* consists of three phases: getting into the *āsana*, holding the *āsana*, and coming out of the *āsana*. Experienced practitioner-teachers are well acquainted with all three phases. Entering the *āsana* involves postural movements requiring simultaneous contraction of multiple muscle groups, including both flexors and extensors, across several joints. In contrast, during the holding phase, practitioners deliberately relax unnecessary muscle activity, particularly in the flexor muscle groups, while maintaining engagement of the extensor muscles, with a conscious emphasis on minimizing muscular effort. This is achieved through precise skeletal alignment that enables efficient load transfer (Figure 1), together with continuous proprioceptive monitoring to progressively reduce the force of muscle contraction (muscular effort), without any deliberate manipulation of breathing. Participants performed the standing asana with such deliberate emphasis on *prayatna-śaithilya* (the reduction of unnecessary muscular and cognitive effort).
3 *Virabhadrasana (E)*: Participants executed the identical external posture of *Virabhadrasana.* They entered the asana in a same way as *Virabhadrasana* (Y) i.e. by simultaneous contraction of multiple muscle groups, including both flexors and extensors, across several joints. However, there was a substantial difference between the holding phase of *Virabhadrasana* (Y) and *Virabhadrasana* (E). In *Virabhadrasana* (E), participants continued to hold the posture by simultaneous contraction of multiple muscle groups, including both flexors and extensors, across several joints resulting into a sustained, isometrically demanding standing postural task without any deliberate emphasis on *prayatna-śaithilya* (the reduction of unnecessary muscular and cognitive effort).

**Figure 1:**
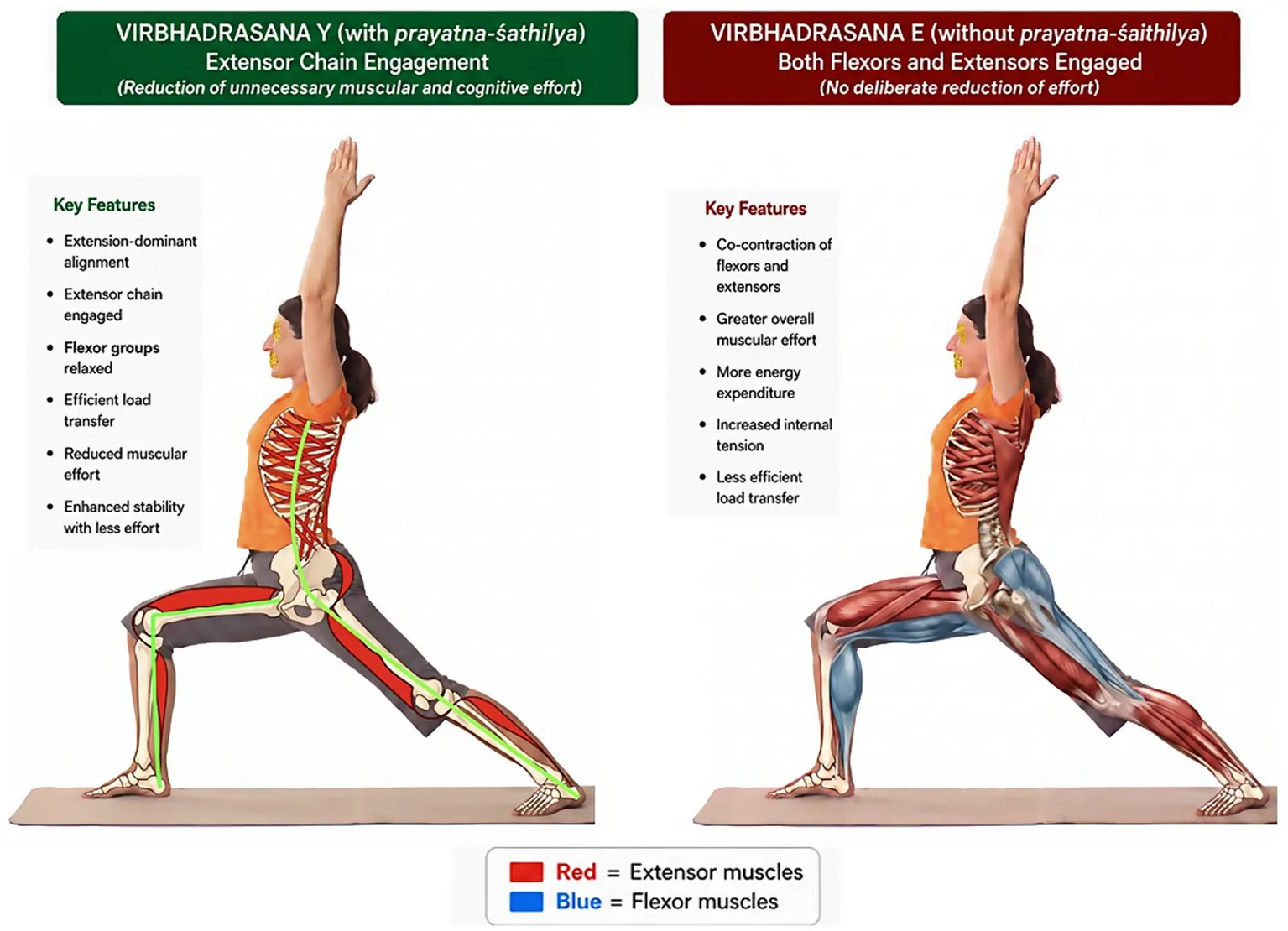
Biomechanical depiction of *Virabhadrasana* (Y, E).

Biomechanical depiction of *Virabhadrasana* (Y) and (E) is given in Figure 1.

Participants were instructed to maintain spontaneous comfortable breathing without paced respiratory control in all experimental conditions. Five-minute rest intervals separated conditions to minimize carryover autonomic effects. Participants completed subjective self-ratings of effort on a Visual Analogue Scale (VAS). A VAS is a standard psychometric tool used in healthcare and research to measure subjective experiences that cannot be directly quantified. It consists of a 10 cm horizontal straight line. Each end of the line has a descriptive anchor representing an extreme of the experience: “Minimum effort” at the left end and “Maximum effort” at the right. The participants were familiar with the VAS and marked the point that best represented their perceived level of effort during the preceding posture immediately following each experimental block.

### 2.3 Data acquisition and ECG processing

Continuous electrocardiographic (ECG) data were acquired throughout the protocol using a two-channel Niviqure ambulatory physiological monitoring system at a sampling rate of 1,000 Hz. Data collection for all participants was completed over two consecutive days between 9:30 a.m. and 12:30 p.m. Raw ECG signals were converted into RR interval (tachogram) time series. The raw tachograms were visually inspected by an expert blinded to the condition hypotheses. Artifacts and ectopic beats were corrected using an adaptive filtering algorithm to ensure that only normal-to-normal (NN) intervals were subjected to subsequent mathematical analyses.

### 2.4 HRV analysis

ECG data from inter-block rest periods were excluded, and only the data obtained during stabilized posture maintenance were used for HRV analysis. Signal stationarity was assessed using segmented analysis. HRV analysis was conducted in accordance with guidelines of the Task Force of the European Society of Cardiology and the North American Society of Pacing and Electrophysiology (Malik, 1996) using validated Chronovisor analysis software (Deshmukh et al., 2024). Median and interquartile range (IQR) values were calculated for RR interval and spectral HRV indices, including total power (TP), low-frequency power (LF: 0.04–0.15 Hz), high-frequency power (HF: 0.15–0.40 Hz), normalized LF and HF values (nu), and LF/HF ratio. Nonlinear HRV measures including sample entropy (SampEn) and detrended fluctuation analysis alpha (DFAα1) were also computed.

### 2.5 Statistical pipeline

Due to the exploratory nature and small sample size of the cohort (N = 8)), non-parametric statistical procedures were strictly utilized.

- *Global testing*: Omnidirectional differences across the three experimental conditions (Free-Standing, *Virabhadrasana* Y and E) were assessed using Friedman’s Two-Way Analysis of Variance by Ranks for each HRV metric.
- *Post-Hoc pairwise comparisons*: post-hoc paired evaluations were conducted using Wilcoxon Signed-Rank Tests.
- *Error correction*: Type I error accumulation from multiple pairwise comparisons were controlled using the Holm–Bonferroni step-down adjustment procedure across pairwise comparison.

Statistical significance for all tests was set *a priori* at (*α* = 0.05). Effect sizes were calculated using rank-biserial correlation (*r*), with values ≥ 0.50 interpreted as large effects. Emphasis was placed on effect sizes and overall physiological patterns rather than isolated *p*-values.

## 3 Results

### 3.1 Descriptive statistics

Median and interquartile range (IQR) values for time-domain, frequency-domain, and nonlinear HRV indices across free-standing, yogic *Virabhadrasana* (Y), and *Virabhadrasana* (E) are presented in Table 1 and Figure 2 (box plots).

**Table 1:** Median and IQR (Q3-Q1) of time and frequency domain and non-linear HRV parameters during performance of free-standing and two modes of *Virbhadrasana* (Y) and (E).

Groups	RR	TP (ms2)	LF(ms2)	HF(ms2)	LF(nu)	HF(nu)	LF/HF	SpEn	DFA alpha
Free-standing	774.06 (741.74–803.37)	1212.57 (914.86–1822,21)	471.83 (343.16–1054.88)	75.54 (41.76–228.05)	87.9 (74.63–91.26)	12.11 (8.74–25.37)	7.4 (3.28–15.27)	1.14 (1.02–1.24)	1.25 (1.09–1.38)
*Virbhadrasana (*Y) (yogic)	713.93 (683.46–774.47)	994.96 (641.7–1498.74)	220.25 (143.78–393.19)	206.08 (78.4–333.53)	62.38 (38.79–77.38)	37.62 (13.22–61.22)	1.74 (0.64–4.46)	1.17 (0.96–1.38)	1.09 (0.93–1.16)
*Virbhadrasana (E)*	637.05 (601.93–719.76)	440.5 (332.59–837.4)	123.75 (85.26–218.99)	28.84 (16.14–83.82)	71.12 (65.2–85.94)	28.88 (14.06–33.31)	2.49 (1.63–6.78)	1.16 (0.98–1.28)	1.21 (1.05–1.46)

RR, RR interval (ms); TP (ms2), Total Power (ms2); LF (ms2), Low frequency absolute power (ms2); HF (ms2), High frequency absolute power (ms2); (nu), Normalized units; LF/ HF, Low frequency to high frequency ratio; SpEn, Sample entropy; DFAα1, Detrended fractal analysis.

**Figure 2:**
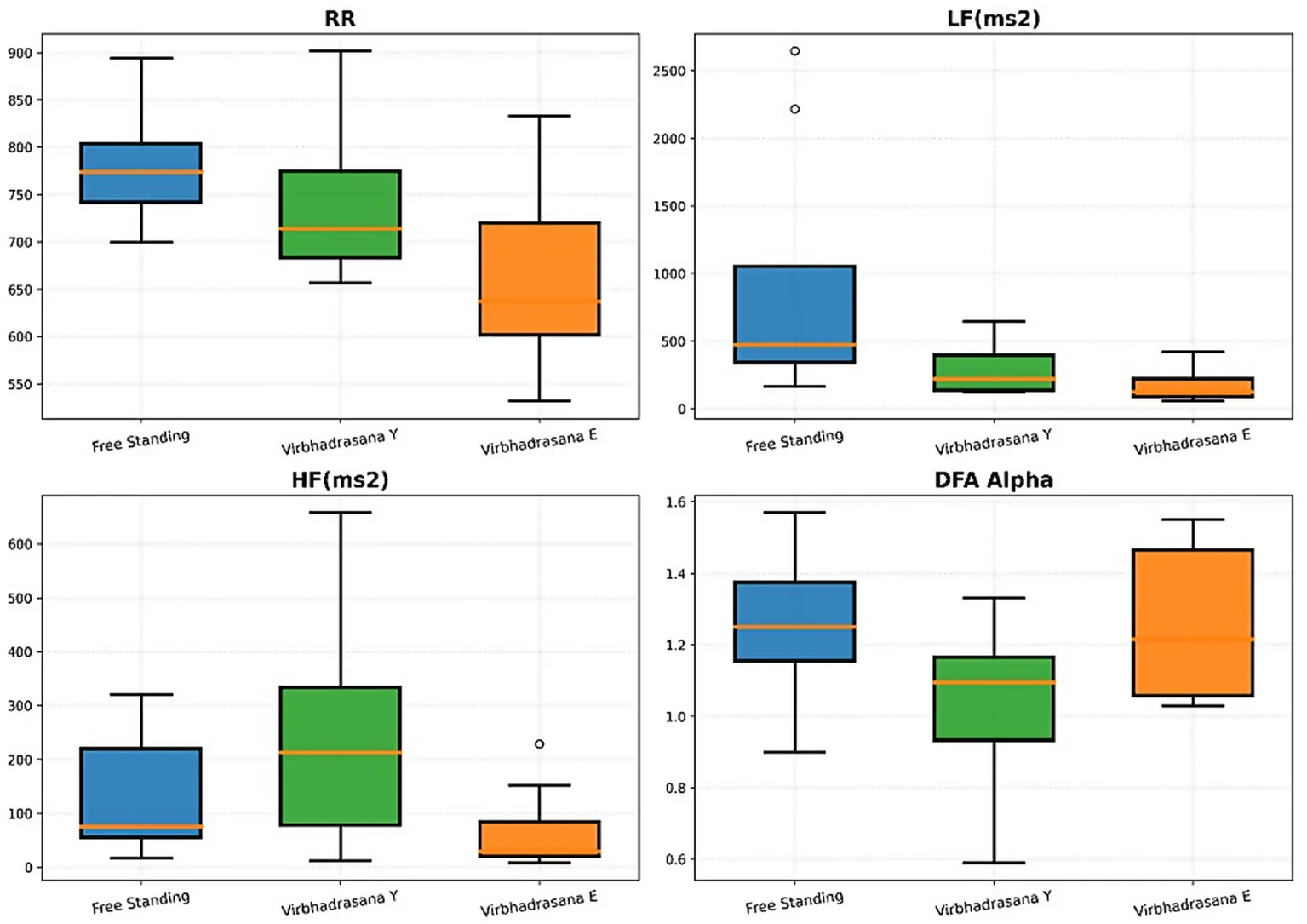
Box plot of time and frequency domain and non-linear HRV parameters during performance of free-standing and two modes of *Virbhadrasana* (Y, E).

Friedman repeated-measures analysis demonstrated significant overall effects of condition on RR interval [*χ*^2^(2) = 10.75, *p* = 0.0046], LF power [*χ*^2^(2) = 7.75, *p* = 0.021], HF power [*χ*^2^(2) = 10.75, *p* = 0.0046], LF (nu) [*χ*^2^(2) = 6.75, *p* = 0.034], HF (nu) [*χ*^2^(2) = 6.75, *p* = 0.034], LF/HF ratio [*χ*^2^(2) = 6.75, *p* = 0.034], and DFAα1 [*χ*^2^(2) = 7.00, *p* = 0.030]. Total power and sample entropy did not demonstrate significant omnibus differences across conditions.

### 3.2 Comparison between *yoga-Āsana* conditions and free-standing

Results of Wilcoxon signed-rank tests and Holm–Bonferroni correction are presented in Table 2.

**Table 2:** Results of wilcoxon signed rank test.

Groups	RR		TP (ms2)		LF (ms2)		HF (ms2)		LF (nu)		HF (nu)		LF/HF		SpEn		DFA	
	*p*-value	*r*	*p*-value	*r*	*p*-value	*r*	*p*-value	*r*	*p*-value	*r*	*p*-value	*r*	*p*-value	*r*	*p*-value	*r*	*p*-value	*r*
Free-standing vs. *Virabhadrasana (*Y) (yogic)	0.19 (0.19)	0.49	0.19 (0.39)	0.49	0.01* (0.03#)	0.84	0.1 (0.2)	0.59	0.03* (0.07)	0.74	0.03* (0.07)	0.74	0.03* (0.07)	0.74	0.19 (0.39)	0.49	0.01* (0.04#)	0.8
Free-standing vs. *Virabhadrasana* (E)	0.007* (0.02#)	0.89	0.19 (0.39)	0.49	0.01* (0.03#)	0.84	0.05* (0.16)	0.69	0.19 (0.19)	0.49	0.19 (0.19)	0.49	0.19 (0.19)	0.49	0.31 0.39)	0.39	0.31 (0.62)	0.39
*Virabhadrasana (*Y) (yogic) vs. *Virabhadrasana* (E)	0.01* (0.03#)	0.84	0.31 (0.39)	0.39	0.07 (0.07)	0.64	0.007* (0.02#)	0.89	0.007* (0.02#)	0.89	0.007* (0.02#)	0.89	0.007* (0.02#)	0.89	0.38 (0.39)	0.34	0.01* (0.03#)	0.84

*significant (uncorrected), Holm-Bonferroni corrected *p*-value given within the parenthesis () # significant after correction.

*Virabhadrasana* (Y) was the deliberate, effort-reduced “yogic” performance, while *Virabhadrasana* (E) was the performance without deliberate effort reduction. Compared with free-standing, *Virabhadrasana* (E) demonstrated a significant reduction in RR interval (median: 774.06 ms to 637.05 ms; *p* = 0.007, Holm–Bonferroni corrected *p* = 0.02, r = 0.89) and significant reduction in LF power (median: 471.83 to 123.75 ms^2^; *p* = 0.01, Holm–Bonferroni corrected *p* = 0.03, *r* = 0.84). HF power showed an uncorrected reduction (median: 75.54 ms^2^ to 28.84 ms^2^; *p* = 0.05), which did not remain significant after Holm–Bonferroni correction (adjusted *p* = 0.16), although the effect size remained large (*r* = 0.69).

In contrast, compared with the free-standing, yogic *Virabhadrasana* (Y) demonstrated a significant reduction in LF power (median: 471.83 ms^2^ to 220.25 ms^2^; *p* = 0.01, Holm–Bonferroni corrected *p* = 0.03, *r* = 0.84), accompanied by increased HF (nu) (median: 12.11 to 37.62 nu; *p* = 0.03, *r* = 0.74) and reduced LF/HF ratio (median: 7.4 to 1.74; *p* = 0.03, *r* = 0.74). Although the statistical significance was attenuated after Holm–Bonferroni correction (adjusted *p* = 0.07), effect sizes remained large (*r* = 0.74).

### 3.3 Between-Āsana comparison

Yogic *Virabhadrasana* (Y) demonstrated the following significant differences compared to *Virabhadrasana* (E):

- Significantly higher RR interval (median: 637.05 ms to 713.93 ms; *p* = 0.01, Holm–Bonferroni corrected *p* = 0.03, *r* = 0.84),
- Significantly higher HF power (median: 28.84 ms^2^ to 206.08 ms^2^; *p* = 0.007, Holm–Bonferroni corrected *p* = 0.02, *r* = 0.89),
- Significantly higher HF (nu) (median: 28.88 nu to 37.62 nu; *p* = 0.007, Holm–Bonferroni corrected *p* = 0.02, *r* = 0.89),
- Significantly lower LF (nu) (median: 71.12 nu to 62.38 nu; *p* = 0.007, Holm–Bonferroni corrected *p* = 0.02, *r* = 0.89),
- Significantly lower LF/HF ratio (median: 2.49 to 1.74; *p* = 0.007, Holm–Bonferroni corrected *p* = 0.02, *r* = 0.89).

For nonlinear HRV indices, DFAα1 was significantly lower in yogic *Virabhadrasana* (Y) compared with both free-standing and *Virabhadrasana* (E) (median: 1.25 and 1.21, respectively, to 1.09; *p* = 0.01, Holm–Bonferroni corrected *p* = 0.03, *r* = 0.80). No significant differences were observed in sample entropy across conditions.

The subjective ratings confirmed the intended distinction between the two modes of execution, with significantly lower perceived effort during yogic *Virabhadrasana* (Y) than during execution of *Virabhadrasana* (E) (median VAS score: 1.5 vs. 6.0; *p* = 0.012; *r* = 0.89). Thus, the VAS findings verified that participants successfully achieved the intended reduction in perceived effort during *Virabhadrasana* (Y).

## 4 Discussion

### 4.1 Divergent autonomic patterns behind identical postures

This exploratory study examined whether externally similar but internally different modes of performing the same standing *Yoga-Āsana* are associated with distinct autonomic regulatory organizations. Our findings indicate that effort-modulated yogic execution of *Virabhadrasana* (Y) preserves vagally mediated adaptive flexibility despite sustained orthostatic and postural demands. Conversely, the identical posture *Virabhadrasana* (E) induces a pattern consistent with generalized autonomic constriction and an increased physiological burden. These observations support the broader hypothesis that physiological cost depends not merely on external task demands themselves, but fundamentally on the internal organization of regulation through which action is achieved.

In this protocol, the free-standing condition represented a typical orthostatic challenge, serving as an active baseline reference against which autonomic responses could be interpreted. During the *Virabhadrasana* (E), participants demonstrated a simultaneous reduction in RR interval, HF power, and LF power. Rather than reflecting sympathetic activation, this pattern indicates a generalized constriction of autonomic regulatory dynamics, characterized by a broad reduction in physiological variability and oscillatory flexibility.

In sharp contrast, the yogic mode *Virabhadrasana* (Y) presented a physiological paradox: an increase in HF activity alongside reduced LF power and a lower LF/HF ratio, despite the ongoing orthostatic and postural demands. When directly compared with *Virabhadrasana* (E), this distinction became even more pronounced, with *Virabhadrasana* (Y) demonstrating significantly higher RR intervals and HF activity, alongside lower LF (nu), lower LF/HF ratios, and decreased DFAα1values. Crucially, these findings point to the preservation of physiological adaptability during sustained, active engagement, rather than a state of passive relaxation.

### 4.2 Efficient regulation reduces physiological cost without reducing task demand

Taken together, these findings support a distinction between constrained and optimized regulation, suggesting that physiological cost depends not only on task demands but also on how regulation is organized (André et al., 2019). Although all conditions imposed broadly similar postural demands, RR interval and HRV organization differed substantially according to the mode of execution. Thus, physiological burden appears to reflect the regulatory cost of sustaining performance rather than the task itself (Friston, 2010; Shenhav et al., 2013). The yogic mode likely represents a lower-cost regulatory strategy that preserves adaptive flexibility despite continued task engagement, whereas the mode without deliberate effort reduction imposes greater physiological burden despite similar external performance (Thayer and Lane, 2000).

This interpretation is consistent with systems neuroscience and motor control, where skilled performance is characterized by optimization rather than maximization of control (Bernstein, 1967). Expertise reduces unnecessary muscular activation, improves coordination, and preserves adaptive reserve while maintaining task demands. Importantly, the more economical autonomic profile observed during yogic execution does not imply passivity or disengagement. Maintaining the posture still required sustained attention, proprioceptive monitoring, and continuous postural adjustment. Instead, the findings suggest an actively regulated yet physiologically economical state in which unnecessary corrective effort and autonomic burden are minimized while effective task engagement is maintained—a state aptly described as “relaxation in action” (Csikszentmihalyi, 1990).

The observations are consistent with Yoga Sutra 2.47, which describes progressive reduction of effort (*prayatna-śaithilya*) during sustained posture, and provide preliminary physiological evidence that this classical principle may correspond to measurable differences in autonomic regulatory organization.

The nonlinear HRV findings further support this interpretation. DFAα1 was significantly lower during the yogic condition than during both free-standing and effort-intensive execution. Since DFAα1 reflects long-range temporal organization and the balance between regulatory rigidity and adaptability (Goldberger et al., 2002; Peng et al., 1995), lower values within the physiological range may indicate a shift toward more flexible, adaptive regulation. Sample entropy did not differ significantly across conditions.

### 4.3 Neurovisceral integration via the central autonomic network—somato-cognitive action network (CAN-SCAN) interface

Although speculative, these findings may be interpreted within the framework of the Central Autonomic Network (CAN), which integrates interoceptive, attentional, emotional, respiratory, and autonomic processes through prefrontal-subcortical circuits, including the ventromedial prefrontal cortex and anterior cingulate cortex, to coordinate adaptive physiological responses (Smith et al., 2017; Thayer et al., 2012). The yogic mode may reflect more efficient real-time integration of posture, breathing, attention, and autonomic regulation reducing regulatory burden while preserving adaptive flexibility.

One possible neural substrate for this efficient co-regulation may be the recently described Somato-Cognitive Action Network (SCAN) (Gordon et al., 2023), which links primary motor regions involved in postural control with the insula and executive components of the CAN. This network provides a plausible architecture for integrating metabolic regulation with goal-directed action.

Within this framework, the autonomic differences observed between yogic and effort-intensive execution may reflect distinct embodied control strategies rather than differences in muscular activation alone. Skilled yogic practice may optimize coordination across posture, respiration, interoception, and autonomic regulation through the CAN–SCAN interface, thereby minimizing the central regulatory cost of maintaining stable performance (Ren et al., 2026).

### 4.4 Pedagogical, clinical and research implications

The findings also have important pedagogical implications. Yoga teaching may benefit from shifting emphasis away from externally imposed postural achievement toward cultivation of efficient regulation and embodied adaptability. In this framework, “good practice” is not defined by maximal stretch, strength, or endurance, but by the ability to sustain action with minimal unnecessary physiological constraint. From a physiological perspective, the “skill” of yoga may therefore lie in learning to maintain postural integrity while minimizing excessive corrective effort and preserving autonomic flexibility. Accordingly, yoga may be understood not simply as exercise or relaxation, but as a training paradigm for optimizing the relationship between effort, regulation, and adaptability.

These observations may also have broader implications for mind–body medicine. Many stress-related conditions and noncommunicable diseases (NCDs) are characterized by autonomic rigidity, reduced vagal flexibility, persistent physiological overcontrol, and impaired adaptive variability (Phadke and Phadke, 2022). The present findings suggest that yoga may exert therapeutic effects not merely through passive relaxation or through the general physiological benefits of physical exercise alone, but possibly by training more efficient modes of physiological regulation during active engagement.

### 4.5 Limitations

Several limitations should nevertheless be acknowledged. The sample size was small and restricted to experienced female yoga practitioner-teachers. The study was exploratory and intended primarily to examine physiological feasibility and conceptual distinctions rather than establish definitive causal conclusions. Holm–Bonferroni correction was applied to all pairwise comparisons within each predefined HRV outcome following a significant Friedman test. We did not apply an additional correction across different HRV variables because these indices are physiologically and mathematically interrelated rather than independent endpoints. Applying a global correction across all HRV measures would substantially increase the risk of Type II error in this small exploratory study. Accordingly, the findings should be interpreted as hypothesis-generating, with emphasis placed on the consistency of physiological patterns and effect sizes in addition to adjusted *p*-values.

Effort was made to follow best methodological practices outlined for psychophysiological research involving HRV (Laborde et al., 2017). Considering that a large inter and intra individual variation may exist in HRV, within-subject design was followed and a cautious interpretation of the HRV indices was adhered to (Goldstein et al., 2011). HRV metrics provide indirect indices of physiological organization and should not be interpreted as isolated markers of discrete sympathetic or parasympathetic activity. Respiratory activity was not independently monitored, and the contribution of respiratory modulation to HF-related findings cannot be fully dissociated. However, respiration itself forms an integral component of embodied autonomic regulation during *Yoga-Āsana* practice rather than a purely external confounding variable. In addition, cortical and muscular activity were not directly measured; therefore, proposed differences in regulatory organization remain inferential.

A strength of the study design was the use of a fixed sequence of experimental conditions, which preserved the natural instructional progression of learning and performing the āsana. This approach minimized the possibility that participants would inadvertently apply the *prayatna-śaithilya* strategy during the control condition, thereby maintaining a clear distinction between *Virabhadrasana* (E) and *Virabhadrasana* (Y). It also reflected the way the intervention is taught and practiced in real-world settings, enhancing ecological validity. Although a fixed sequence may introduce potential order effects, including learning, carryover, fatigue, habituation, or expectation, preserving the integrity of the intervention was considered the higher methodological priority in this proof-of-concept study. Future studies may incorporate randomized or counterbalanced designs to examine whether these findings are robust to different condition orders.

Future studies incorporating respiratory monitoring, electromyography, metabolic assessment, neurophysiological recordings, and longitudinal training designs may help further clarify the mechanisms underlying the observed autonomic differences. Despite the exploratory nature of the study and its methodological limitations, several observations survived Holm–Bonferroni correction, and the convergence of large effect sizes across conditions permits meaningful interpretation.

### 4.6 Conclusion

This study provides preliminary physiological proof-of-concept that externally similar but effortfully different postural behaviors (*Virbhadrasana* Y and E) were associated with different autonomic (HRV) patterns in a small group of experienced practitioners that need to be replicated in a larger sample. By operationalizing the classical yogic principle of effort reduction (*prayatna-śaithilya*), the results support the broader hypothesis that efficient regulation is characterized by preservation of adaptive flexibility alongside reduction of unnecessary physiological expenditure during sustained action. In this sense, skilled *Yoga-Āsana* may provide a useful experimental paradigm for investigating efficient embodied regulation and the neurophysiology of effort during sustained action. This also highlights the utility of deliberate effort modulation in distinguishing *Yoga-Āsana* from ordinary exercise and supports further investigation of its potential therapeutic role in stress-related disorders and non-communicable diseases (NCDs).

## Data Availability

The raw data supporting the conclusions of this article will be made available by the authors, without undue reservation.
